# A Rare Case of Concurrent Intraventricular Meningioma and Choroid Plexus Papilloma: A Case Report

**DOI:** 10.3390/medicina60122100

**Published:** 2024-12-22

**Authors:** Daniel Markov, Kristian Bechev, Usman Khalid, Vladimir Aleksiev, Galabin Markov, Elena Poryazova

**Affiliations:** 1 Department of General and Clinical Pathology, Medical University of Plovdiv, 4002 Plovdiv, Bulgaria; kristian_bechev@abv.bg (K.B.); eporiazova@abv.bg (E.P.); 2 Department of Clinical Pathology, University Multidisciplinary Hospital for Active Treatment (UMHAT) “Pulmed”, 4002 Plovdiv, Bulgaria; 3 Neurological Surgery, Pulmed Univeristy Hospital, 4000 Plovdiv, Bulgaria; 4 Faculty of Medicine, Medical University of Plovdiv, 4002 Plovdiv, Bulgaria; usmankhalid957@gmail.com (U.K.); gabi_markov@abv.bg (G.M.); 5 Department of Thoracic Surgery, UMHAT “Kaspela”, 4002 Plovdiv, Bulgaria; vl_alex@abv.bg; 6 Department of Cardiovascular Surgery, Medical University of Plovdiv, 4002 Plovdiv, Bulgaria

**Keywords:** meningioma, choroid plexus papilloma, intraventricular tumors

## Abstract

This case presents an unusual combination between an intraventricular meningioma and a choroid plexus papilloma. Intraventricular meningiomas are rare intraventricular tumors presenting with symptoms of hydrocephalus, headache, and neurological deficits. The rarity of choroid plexus papillomas is highlighted in medical diagnostics, with the majority of these findings being incidental within the setting of obstructive hydrocephalus. We present the case of a 66-year-old male patient who exhibited symptoms of headaches. Magnetic resonance imaging showed the presence of an intraventricular tumor in the region of the trigone of the right lateral ventricle, which was assessed for surgical removal. A postoperative hemorrhage necessitated a reoperation, after which the patient began to gradually recover. Hemianopsian disturbances persisted during and after his hospital stay. This report describes a rare case of concurrent choroid plexus papilloma and ventricular meningioma, manifesting as a single tumor mass.

## 1. Introduction

Meningiomas comprise the largest group of tumors in the central nervous system. Meningeal tumors arise from the meningothelial cells that line the meninges. Benign variants of meningiomas predominate in adults, with the female sex being more commonly affected. The most frequent location of occurrence is the fourth ventricle, with the lateral and third ventricles being less commonly involved. The incidence of intraventricular meningiomas is relatively low compared to other types of brain tumors, accounting for approximately 0.5–5% of all meningiomas [1]. Despite this, their incidence may fluctuate depending on a combination of factors, including age, gender, and geographic location.

Meningiomas often present with clinical symptoms of brain mass lesions such as seizures, focal neurological deficits, or headaches, depending on their anatomical location and size. However, asymptomatic cases or those with non-specific symptoms may also occur.

We report an uncommon case of concurrent intraventricular meningioma and choroid plexus papilloma, a rare combination with distinctive pathological origins. Intraventricular meningiomas derive from the meninges, while choroid plexus papillomas originate from specialized epithelial cells of the choroid plexus, responsible for cerebrospinal fluid production. This juxtaposition of two different tumor entities poses notable challenges in their diagnostic and therapeutic management. We detailed the clinical report, radiological manifestations, histopathological features, and therapeutic strategies.

This case underscores the exigency for high awareness regarding the rare tumor co-occurrence within the intraventricular space, as this type of combination of intraventricular meningioma mixed with choroid plexus papilloma is extremely rare, and there are only isolated reports in the available literature. This provides insight into the rarity of such coexisting benign tumors and highlights the need for careful diagnostic evaluation.

## 2. Case Presentation

### 2.1. Clinical Features and Diagnostic Imaging Results

**Figure 1:**
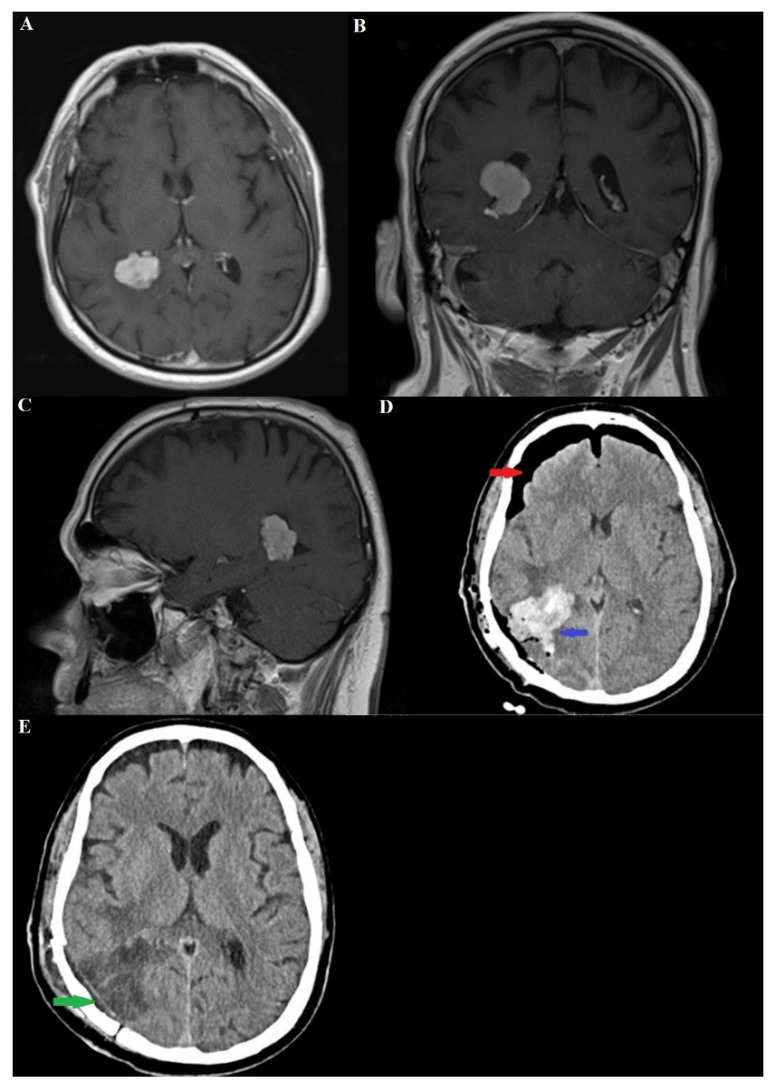
(**A**–**C**) MRI of the brain with contrast material shows a well-defined and intensely saturated tumor mass located in the region of the trigonum of the right lateral ventricle, presented in axial, coronal and sagittal planes with dimensions: 2.44/2.06 cm; 2.28/2.32 cm; 2.00/2.46 cm. (**D**) Postoperative brain CT showing intraparenchymal cerebral hemorrhage (blue arrow) extending to the ventricular system associated with frontal pneumocephalus (red arrow). (**E**) Postoperative brain CT scan eight days after surgery shows ischemic changes in the brain parenchyma and perifocal ischemic edema (green arrow).

A 66-year-old man presented with a history of headaches for the past few months. The pain was described as a dull, intermittent ache that responded to analgesics and was not limited to one side of the head. Three months before the neurosurgical diagnosis, a transurethral tumor was histologically verified, for which treatment had been started in the oncology department. The accompanying diseases of the patient were as follows: arterial hypertension, ischemic heart disease, Hashimoto’s thyroiditis. Neurological examination showed no evidence of sensory, motor, or cranial nerve deficits. Up to this point, the patient had no history of stroke or family history of cancer. On the occasion of the persistent headache, a magnetic resonance imaging (MRI) of the brain was performed, revealing a well-differentiated and contrast-enhancing mass in the area of the trigone of the right lateral ventricle with the dimensions of the axial, coronal and sagittal sections as follows: 2.44/2.06 cm; 2.28/2.32 cm; 2.00/2.46 cm Figure 1A–C.

Imaging procedures initially indicated obstructive dilation of the occipital and temporal horns of the right lateral ventricle. The differential diagnoses included intraventricular meningioma, choroid plexus papilloma, and exophytic intraaxial tumors. Total surgical extirpation of the tumor was selected as the treatment approach, utilizing a right parieto-occipital craniotomy with Keene’s point as the central landmark for the bony trepanation. Ultrasonographic verification of the surgical corridor to the trigone of the lateral ventricle was done. A transgyral approach was used to reach the tumor formation. The latter was devascularized from its feeding vessels, branches of the posterior choroid artery being of primary importance. Total extirpation of the tumor mass followed.

A few hours later, after extubation, the patient developed an obscuration of consciousness from marked soporosity to coma. The Glasgow Coma Scale (GCS) score reached 7 points, which necessitated a follow-up computer tomography (CT) (Figure 1D,E). On the latter, a parenchymal brain hemorrhage combined with a ventricular one (at the site of the tumor bed) was detected. A repeat operation followed in order to evacuate the hematoma contents. No additional hemorrhagic incidents were detected at subsequent CAT controls, and no development of additional dilatation of the ventricular system or formation of hydrocephalus was observed.

### 2.2. Pathological Examination

The intraventricular tumor was observed to have a grayish-whitish color with a fine granular structure. It presented as a rounded mass measuring 2.5 by 2 by 1 cm, with a vascular bundle 3 cm long. The tumor had an uneven surface and a dense consistency with a whitish color upon cut-sectioning.

Histological examination revealed an abundance of meningothelial cells and psammoma bodies. Additionally, features of choroid plexus papilloma were observed within the same tumor mass. Morphological diagnosis identified the tumor as an intraventricular meningioma coexisting with choroid plexus papilloma.

Immunohistochemical examination showed GFAP negativity, EMA–focal positivity, CK negativity, and a Ki-67 index of less than 2%.

**Figure 2:**
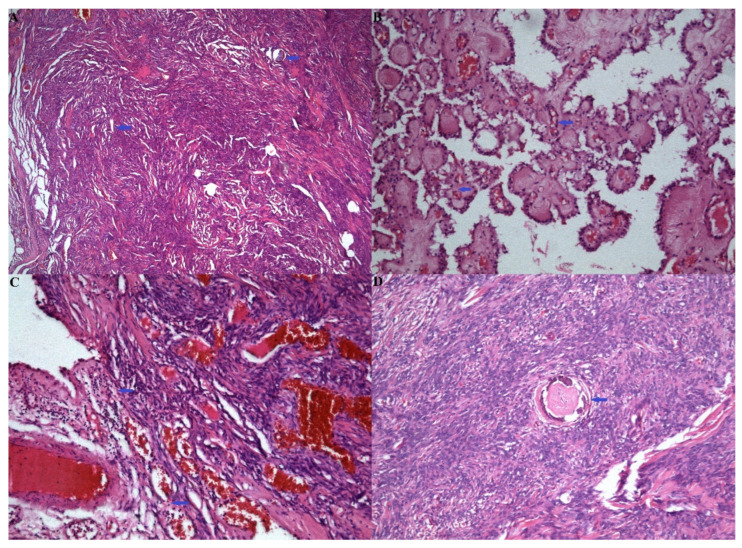
(**A**) Meningeal cells, among which are found single preserved psammoma bodies—the blue arrow points at psammoma bodies. (H.E. ×25); (**B**) Choroid plexus papilloma with preserved papillary architecture, with fibrovascular cores, lined by one layer of cuboidal epithelium—the blue arrow points at papillary structures. (H.E. ×100); (**C**) Zone of transition between the meningeomatous and papillomatous components—the blue arrow points at the zone of transition. (H.E. ×100); (**D**) Psammoma body within the tumor parenchyma—the blue arrow points at psammoma body. (H.E. ×100).

The histological and immunohistochemical findings confirmed the diagnosis of an intraventricular meningioma with choroid plexus papilloma (Figure 2).

### 2.3. Clinical Course

The patient underwent a 12-day hospitalization, followed by ongoing monitoring with periodic assessments and CT imaging for continued evaluation. Three months after the surgical treatment, the patient was clearly conscious, communicative and adequate, without sensory and motor neurodeficiency. Despite this, bitemporal hemianopsia persisted. No recurrence of the tumor was detected from the follow-up MRI, therefore no subsequent radiotherapy was required.

## 3. Discussion

Choroid plexus tumors comprise a wide range of neoplasms, from well-differentiated meningiomas classified as WHO grade I to highly anaplastic meningiomas categorized as WHO grade III. The fourth ventricle is the most frequent location for these tumors, followed by the lateral and third ventricles. In children, tumors are more commonly found in the lateral ventricles, whereas in adults, the fourth ventricle is the predominant site. Lower-grade tumors, according to WHO classification, are typically more prevalent in the fourth ventricle, while anaplastic variants are more often observed in the lateral ventricle [2]. According to histological data, meningiomas represent about 36.6% of all benign brain tumors. The overall incidence of meningiomas was about 8.3 per 100,000 people between 2010 and 2014, and data show that it has increased in recent years [3]. The incidence of the disease increases with age, with a 1:3 incidence of intracranial meningioma in males versus females [4]. Choroid plexus meningiomas account for about 0.5–2% of all intracranial meningiomas, and the presence of two separate tumor processes in a single tumor mass is extremely rare, and only single clinical cases have been reported in the literature [5]. Choroid plexus tumors represent different variants, from benign papillomas classified as WHO grade I to carcinomas presenting as WHO grade III. Literature evidence suggests that choroid plexus tumors are relatively rare and range between 0.4–0.6% of the total percentage of primary tumors affecting the central nervous system (CNS) [2].

Among all primary intracranial neoplasms, meningiomas are known for their tendency to coexist with other types of tumors. This phenomenon may be due to the typically indolent nature of meningiomas, which permits a prolonged clinical evolution prior to diagnosis. Furthermore, the detection of meningiomas is often incidental due to their slow growth, and very often, they debut with neurological symptomatology months after the first symptom [6].

Intraventricular meningiomas and choroid plexus papillomas typically present with subtle symptoms that vary with tumor size and location, often causing delayed diagnosis. Common signs include progressive mental status decline, headaches, visual disturbances, hydrocephalus, vomiting, and occasionally, lateral gaze palsy—all indicative of increased intracranial pressure. Despite this, our patient only presented with dull, intermittent headaches, emphasizing non-specific onset of this pathology [7,8].

A number of cases of choroid plexus papilloma in combination with meningioma have been described, but despite this, no unified theory explains the occurrence of synchronous tumors with such localization [5]. Several studies propose that both tumors may originate simultaneously from common progenitor cells, while others suggest that the presence of one tumor may promote the development of the other. At this stage, it remains unclear which of the two tumors typically arises first [2,5,6]. (Table 1).

Most meningiomas are sporadic, but some are associated with genetic syndromes or mutations. Meningiomas, even the sporadic ones, have a mutation in chromosome 22 (Merlin tumor suppressor gene, SIS oncogene or INI1). The same mutation is seen in neurofibromatosis type 2 (NF2), also called MISME syndrome, leading to multiple hereditary schwannomas, meningiomas and epidemics. Loss of chromosome 22q occurs in 89% of intraventricular meningiomas and loss of chromosome 1p in 44%. NF2 mutations are the most common genetic alterations found in intraventricular meningiomas [3]. Data suggest that type II neurofibromatosis should manifest at a younger age, whereas in our patient at 66 years old, the disease was potentially rooted in genetic mutations triggered by hormonal influences or ionizing radiation, which acted as primary risk factors in tumorigenesis.

The combination of choroid plexus papilloma and meningioma in a single tumor mass can complicate the diagnosis during pathological examinations. The diagnostic workflow consists of clinical, cross-sectional imaging and histological studies. Key histologic differential diagnoses encompass a spectrum of tumors, including papillary meningioma, papillary ependymoma and metastatic carcinoma with papillary features [5,11]. The perivascular pseudopapillary pattern accounts for the majority of papillary meningioma, with the diagnosis of papillary meningioma being definitive once supported by an aggressive clinical presentation alongside high-grade histologic features [12]. Despite both papillary ependymomas and choroid plexus papillomas having a papillary structure with a fibrovascular core, the finger-like growth projection lined by single or multiple layers of cuboidal cells with smooth contiguous surface is a key histologic finding of papillary ependymomas [11]. Furthermore, the rough hobnail appearance of a choroid plexus papilloma coupled with a lack of extensive glial fibrillary acidic protein immunoreactivity holds value in further differentiating the two tumors [4,13].

The mechanism behind the occurrence of primary brain tumors colliding at the same site, particularly those with mixed components of choroid plexus papilloma and meningioma, remains unclear. Various theories have been proposed to explain the presence of concurrent primary intracranial tumors [5,11]. Several theories have been suggested: (1) distinct tumors may collide at the same location; (2) an initial tumor might stimulate local growth factors, leading to a secondary tumor; (3) bidirectional differentiation from common progenitor cells could result in mixed tumor elements; and (4) commonly occurring tumors might coincidentally develop together. Although these hypotheses offer some insights, none have been definitively validated [5,12]. Additional research and further case studies are essential to deepen our understanding of the mechanisms driving the concurrent development of multiple intracranial neoplasms [14,15].

MRI of the brain plays a central role in the diagnosis of meningiomas and has become a cornerstone in recent years for both identifying these tumors, regardless of location and planning surgical resection. A hallmark of meningiomas is the presence of a “dural tail”, a distinctive feature that highlights their attachment to the meninges. In our case, however, the tumor demonstrates a connection with the choroid plexus. On T2-weighted imaging, the lesion exhibits a primarily isointense, occasionally hypointense signal. Following gadolinium contrast administration, the tumor appears hyperintense on T2-weighted sequences, with the contrast enhancement also delineating the vascular supply to the tumor [16,17,18].

Intraventricular tumors, regardless of their histological type, present a formidable neurosurgical challenge due to their deep-seated location near critical functional regions. This positioning requires a meticulous and often staged approach to microsurgical devascularization and resection to minimize risks. In cases like ours, where a histologically confirmed meningioma of the choroid plexus coexists with a choroid plexus papilloma, the neurosurgical goal is complete tumor removal to enhance patient survival and quality of life. If complete resection is not feasible, adjuvant radiotherapy may be considered to manage residual tumors, as additional surgical manipulation may carry significant risks. Neuronavigation based on preoperative MRI plays a major role in surgical interventions, as well as an individual approach in the case of a conscientious patient [2,3,17,19,20].

## 4. Conclusions

The admixing of two histological types of tumors is an extremely rare clinical and pathological finding and mostly suggests their simultaneous occurrence in the cerebral ventricle, such as the combined choroid plexus papilloma and meningioma. An atypical case like this requires precise preoperative diagnosis via MRI and histological verification. Surgical resection remains the primary treatment approach, with the goal of complete extirpation removal to optimize patient outcomes. Given the histological diagnosis and the low proliferation index of the tumor cells, subsequent radiotherapy was deemed unnecessary. Clinical follow-up with follow-up MRI scans remains the gold standard. This case report highlights the importance of considering the possibility of rare coexisting pathologies in patients with brain lesions, focusing on the need for comprehensive diagnostic work to ensure accurate diagnosis and precise management.

## Figures and Tables

**Table 1:** Comparison with identical rare cases.

Case	Demographics	Clinical Features	Tumor Details	Continuity	Treatment/Prognosis
Case 1 Ref. [9]	45/F	Headache for 2 months, sensory and motor deficits	Choroid plexus papilloma—Petrotentorial junction—2.5 cm; Meningioma—Cervicomedullary junction—5 cm	Noncontiguous	Surgical resection; good recovery
Case 2 Ref. [10]	45/F	Progressive confusion for 2 months, sensory/motor deficits	Choroid plexus papilloma—Fourth ventricle—4.4 cm; Meningioma—Craniocervical junction—2.3 cm	Noncontiguous	Surgery performed; neurological improvement
Case 3 Ref. [5]	42/M	Headache for 2 weeks, no neurological deficits	Choroid plexus papilloma and meningioma—Lateral ventricle—3 cm	Contiguous	Combined resection; gamma knife radiosurgery for remnant at 3-month follow-up
Case 4 [Present case]	66/M	Persistent headache for 2+ months, no neurological deficits	Choroid plexus papilloma and meningioma—trigone of the right lateral ventricle—2.45 cm	Contiguous	Total surgical resection; treatment for transurethral tumor initiated

## Data Availability

The authors declare that the data supporting the findings of this study are available within the article.
